# 

**Published:** 1855-09

**Authors:** 


### CONTENTS.

### ORIGINAL COMMUNICATIONS.

PAGE.

ARTICLE I.—Medicine as It Is. An Essay. By JOSEPH P. LOGAN, M. D., Professor of Physiology and General Pathology in the Atlanta Medical College, ........ 1

ARTICLE II.—A Case of Parturition, with its Complications, &c. By E. F. KNOTT, M. D., of Griffin, Georgia........ 11

ARTICLE III.—A Report from the Case Book of THOS. S. POWELL, M. D., Sparta, Georgia........ 15

ARTICLE IV.—Dislocation of the Knee Joint, with Fracture of the Patella. By B. O. JONES, M. D., Atlanta, Georgia........ 17

ARTICLE V.—Intense Cold as a Local Anæsthetic. By D. W. HAMMOND, M. D., Macon, Georgia........ 20

ARTICLE VI.—Reports from the Case Book ot J. JUNIUS HARRIS, M. D., and HORATIO N. HOLLIFIELD, M. D., Tennille, Georgia........ 21

ARTICLE VII.—Report of the Surgical Clinic of the Atlanta Medical College. By W. F. WESTMORELAND, M. D., Professor of the Principles and Practice of Surgery........ 24

ARTICLE VIII.—An Oleaginous Element Indispensable in Phthisis. By J. A. DAMOUR, M. D., Macon, Georgia........ 28

### SELECTIONS.

On Diseases of Infancy; their Importance and Fatality........ 30

Case of Nephritic Colic, with Remarks........ 33

Three Sugical Cases........ 35

*Medical Jurisprudence.*—Prolonged Retention of Life by Infants who have not breathed........ 38

The Pathology and Treatment of Leucorrhœa........ 40

A great Medical Discovery—Mercury taken from the system by Electricity.... 49

External Use of the Acid Nitrate of Mercury........ 51

A very Creditable Case—Life saved by the Doctor........ 52

Napor of Iodine in Mammary Tumors........ 53

Mr. Syme on Chloroform........ 54

Medical Periodicals........ 54

Editorial and Miscellaneous........55

